# Anomalous origin of right coronary artery with interarterial course revealed by effort angina: case report

**DOI:** 10.11604/pamj.2021.38.327.26266

**Published:** 2021-04-06

**Authors:** Hind Regragui, Badre El Boussaadani, Chakib Benajiba, Hanae Bouhdadi, Hicham Wazaren, Mohammed Cherti

**Affiliations:** 1 Department of Cardiology B, Mohammed V University, Ibn Sina University Hospital Center, Rabat, Morocco,; 2 Department of Cardiovascular Surgery A, Mohammed V University, Ibn Sina University Hospital Center, Rabat, Morocco

**Keywords:** Right coronary artery, interarterial course, congenital heart disease, myocardial ischemia, case report

## Abstract

Anomalous origin of coronary artery with interarterial course is recognized as a rare congenital heart disease. Its main manifestation is myocardial ischemia related to systolic compression of coronary arteries positioned between the great arteries. We report a case of a middle-aged man admitted in our department for an effort angina during nordic walking. A coronary angiography was performed showing an anomalous coronary artery with a birth defect giving a right common trunk of the circumflex artery and the right coronary artery. We proceeded to a multidetector computed tomography coronary angiography (MDCTCA) to describe this variant of an anomalous coronary arteries which revealed a birth defect in the left anterior sinus of the right coronary and the circumflex artery from a right common trunk passing between the aorta and the trunk of the pulmonary arteries. Magnetic resonance imaging (MRI), exercise stress test and myocardial perfusion scintigraphy were performed in order to objectify an ischemia. Despite the positivity of myocardial scintigraphy, we recommended to our patient to limit exercise with a regular follow-up since he is only symptomatic during a major effort. There are many types of anomalous coronary arteries and the anatomic variant of a right coronary artery that course between the great vessels represents a risk of adverse event and sudden death in young athletes. The choice of therapy is controversial and depends especially on the variant of anomalous coronary artery and the symptoms.

## Introduction

Anomalous origin of right coronary artery (RCA) arising from the left coronary cusp that courses between the great vessel is a rare congenital heart disease that occurs between 0.026% and 0.250% in general population [1]. Clinical presentation is variable and generally related to myocardial ischemia which results from systolic compression of coronary arteries positioned between the great arteries. Here in we report a case of a middle-aged man who presents an effort angina during nordic walking. The diagnosis of anomalous or right coronary artery was made by both coronary angiography and multidetector computed tomography coronary angiography (MDCTCA). The choice of therapy is controversial. In our case, we recommended to the patient to limit exercise with a regular follow-up since he is only symptomatic during a major effort.

## Patient and observation

**Figure 1:**
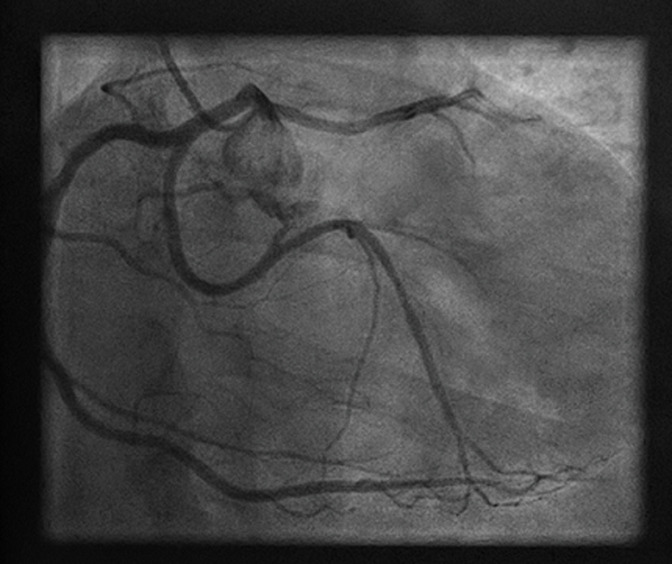
coronary angiography showing an anomalous origin of coronary artery with a right common trunk of the circumflex artery and the right coronary artery

**Figure 2:**
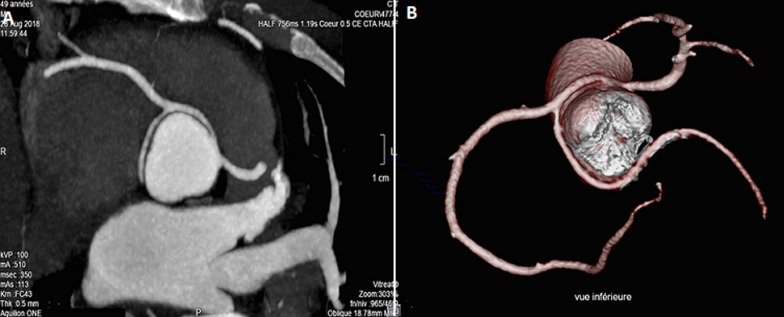
CT coronary artery, A) transverse view showing anomalous origin of right coronary artery with interarterial course (the birth of a common trunk from the left anterior sinus passing between the aorta and the pulmonary artery and giving right coronary artery and circumflex artery); B) inferior view of a three-dimensional reconstruction demonstrating arterial paths

A 49-years-old man, without any cardiovascular risk factor, who presents an effort angina during nordic walking was admitted to our department. Clinical examination and electrocardiogram hadn´t shown any abnormalities. An exercise stress test was carried and was electrically positive, with a 2mm ST segment depression in the left precordial leads at maximum stress (210 Watt and 90% of maximum heart rate). A coronary angiography was performed and had shown an anomalous coronary artery with a birth defect giving a right common trunk of the circumflex artery and the right coronary artery (Figure 1). As part of the coronary assessment in order to specify the coronary paths, a MDCTCA was performed and had demonstrated a variant of an anomalous coronary arteries with birth defect in the left anterior sinus of the right coronary and the circumflex artery from a right common trunk passing between the aorta and the trunk of the pulmonary arteries (Figure 2). Myocardial perfusion scintigraphy was performed and had shown signs of antero-apical myocardial ischemia in 4 segments without sequelae of necrosis then we performed a stress MRI that hadn´t shown any signs of myocardial ischemia. Considering the mismatch between these 2 stress tests and after consultation meeting with cardiovascular surgeon, we recommended to our patient to limit exercise with a regular follow-up since he is only symptomatic during a major effort.

## Discussion

Anomalous origin of the RCA arising from the left coronary cusp that courses between the great vessels occurs between 0.026% and 0.25% in general population and 3 types of have been described [1]. The position between the aorta and the pulmonary artery puts the coronary arteries at risk for compression particularly during strenuous activity and may significantly limit the reservoir capacity of the epicardial coronary system [2]. In addition, sharp angulation of the coronary artery at its origin from the opposite sinus, causes an acute closure in the ostium of the coronary artery responsible for the symptoms [3]. The onset of symptoms probably occurs after exceeding the ischemic threshold, when the increasing size of the aorta such as in sportsmen during hard activities [3], or pulmonary artery combines with an increased adrenergic surge to produce mechanical compression of the anomalous coronary artery [1]. Clinical presentation is variable ranging from the asymptomatic patient to symptoms occurring during exercise including effort angina, dyspnea, arrhythmias or syncope, and at worst acute myocardial infarction or sudden death in the absence of atherosclerosis. Diagnosis can be made coronary angiography but MDCTCA is the « gold standard » for the evaluation of coronary anomalies [4]. Exercise stress test and myocardial perfusion scintigraphy should be used if atypical clinical presentation in order to relate symptoms to the pathology and guide the management. The choice of therapy is controversial with 3 options: medical treatment, coronary angioplasty with stenting and surgical repair [5]. Coronary angioplasty with stenting had been described [6] as a successful method with improved symptoms at >1-year follow-up in 71% of cases and the stent restenosis rate was 13% at a mean follow-up time of 5 years. Several surgical methods have been reported [7]. However, transaortic modification of the origin and proximal portion of the ectopic or anomalous RCA is preferred because it addresses all the mechanisms that cause ischemia, including systolic compression of the trunk [7]. In Japan, 56 patients with anomalous origin of coronary artery who were treated medically with strenuous activity restriction, there was no death related to the anomalous coronary artery during 5 years of follow-up [8].

## Conclusion

Anomalous origin of right coronary artery with interarterial course is a rare congenital heart disease that can present with symptoms similar to coronary artery disease, and sudden cardiac death. The diagnosis is made preferentially by MDCTCA. The choice of therapy is controversial and depends especially on the variant of anomalous coronary artery and the symptoms. Watchful observation and strenuous activity restriction were applied in our case since he is only symptomatic during a major effort.
